# Fabrication of pH/Redox Dual-Responsive Mixed Polyprodrug Micelles for Improving Cancer Chemotherapy

**DOI:** 10.3389/fphar.2021.802785

**Published:** 2022-02-03

**Authors:** Ji Luo, Shuguang Zhang, Peiyao Zhu, Wenke Liu, Jiang Du

**Affiliations:** Department of Thoracic Surgery, The First Affiliated Hospital of China Medical University, Shenyang, China

**Keywords:** polyprodrug, drug delivery, pH/redox responsive, controlled release, cancer chemotherapy

## Abstract

In this work, we prepared pH/redox dual-responsive mixed polyprodrug micelles (MPPMs), which were co-assembled from two polyprodrugs, namely, poly(ethylene glycol) methyl ether-*b*-poly (*β*-amino esters) conjugated with doxorubicin (DOX) *via* redox-sensitive disulfide bonds (mPEG-*b*-PAE-*ss*-DOX) and poly(ethylene glycol) methyl ether-*b*-poly (*β*-amino esters) conjugated with DOX *via* pH-sensitive *cis*-aconityl bonds (mPEG-*b*-PAE-*cis*-DOX) for effective anticancer drug delivery with enhanced therapeutic efficacy. The particle size of MPPMs was about 125 nm with low polydispersity index, indicating the reasonable size and uniform dispersion. The particle size, zeta-potential, and critical micelle concentration (CMC) of MPPMs at different mass ratios of the two kinds of polyprodrugs were dependent on pH value and glutathione (GSH) level, suggesting the pH and redox responsiveness. The drug release profiles *in vitro* of MPPMs at different conditions were further studied, showing the pH—and redox-triggered drug release mechanism. Confocal microscopy study demonstrated that MPPMs can effectively deliver doxorubicin molecules into MDA-MB-231 cells. Cytotoxicity assay *in vitro* proved that MPPMs possessed high toxic effect against tumor cells including A549 and MDA-MB-231. The results of *in vivo* experiments demonstrated that MPPMs were able to effectively inhibit the tumor growth with reduced side effect, leading to enhanced survival rate of tumor-bearing mice. Taken together, these findings revealed that this pH/redox dual-responsive MPPMs could be a potential nanomedicine for cancer chemotherapy. Furthermore, it could be a straightforward way to fabricate the multifunctional system basing on single stimuli-responsive polyprodrugs.

## Introduction

In clinical practice, chemotherapy is still one of the most common strategies for cancer treatment (Galluzzi et al., 2015; Srinivasan et al., 2018; Men et al., 2019), although some emerging therapies like immunotherapy have been recently raised (Chen et al., 2016; Nam et al., 2019; Wang et al., 2019; Yan et al., 2019). However, some drawbacks like high side effect and low bioavailability limit the wide applications of chemical anticancer drugs (e.g., doxorubicin, paclitaxel, and camptothecin), which possess high anticancer activity (Dai et al., 2016; Saravanakumar et al., 2016; Gao et al., 2018). For example, doxorubicin hydrochloride (DOX-HCl), widely used in clinics, can induce severe cardiotoxicity, leading to severe reverse effects and poor therapeutic efficiency due to the low bioavailability (Shabalala et al., 2017; Mao et al., 2019).

To overcome these obstacles, drug delivery systems (DDSs), which could be a promising approach to address these issues, have attracted more and more attention in these decades (Bauri et al., 2018; Talebian et al., 2018). Particularly, stimuli-responsive block copolymers have been thoroughly investigated and extensively used as nanocarriers for drug delivery in biomedical fields, especially cancer therapy (John et al., 2015; Hu et al., 2017; John et al., 2017; Guo et al., 2020). Some specific physiological characteristics in tumor microenvironment (TME) compared to the normal human environment, such as low pH, high glutathione (GSH) concentration, and special enzyme, could be used as trigger cues for drug controlled release (John et al., 2016; Gao and Lo, 2018; Saw et al., 2019; He et al., 2020; Yang et al., 2020). For instance, Li et al. developed a pH/redox dual-responsive hybrid polymer–lipid system (HDPLNPs) for drug delivery and controlled release in cancer chemotherapy. The anticancer drug doxorubicin (DOX) was physically loaded in the core of the nanoparticle, resulting in dual stimuli-responsive system (DOX-HDPLNPs). The physically loaded drug DOX molecules would be released by responding to the acidic pH and high GSH level after deposition at tumor site. Compared to the free DOX formulation, the DOX-HDPLNPs showed much higher tumor inhibition efficiency and lower side effect *in vitro* and *in vivo* (Li et al., 2018). Kim and co-workers synthesized a series of dual stimuli-responsive triblock copolymers based on pH-sensitive poly(l-histidine) segment and redox-sensitive disulfide linker for improving delivery of DOX in cancer chemotherapy. The results showed that DOX-loaded system had high drug loading content with enhanced DOX release in an acidic environment in the presence of 10 mM glutathione. All the results demonstrated that this system could effectively inhibit the CT26 tumor (John et al., 2017). Tang et al. prepared novel pH/redox dual-sensitive platinum (IV)-based micelles to improve the therapeutic efficacy for combination chemotherapy *in vitro* and *in vivo* (He et al., 2019). Wu et al. reported a novel pH/redox-sensitive block copolymer, which was synthesized by polycondensation of disulfide-bond-containing dimethyl cystinate and polycaprolactone oligomer *via* a pH-responsive imine bond for anticancer drug paclitaxel (PTX) delivery in enhancing cancer chemotherapy. The results showed that the PTX-loaded system exhibited an excellent tumor-inhibiting ability and good drug tolerability (Zhang et al., 2019a). Recently, various multifunctional polymeric carriers conjugated with chemical drug molecules have been developed as polyprodrug to improve the therapeutic efficacy and reduce the cytotoxicity (Hu and Liu, 2017; Xu et al., 2017; Zhang et al., 2019b; Wang et al., 2020). For instance, Xiao et al. reported a polyprodrug based on poly(disulfide) conjugated with DOX on the polymer side chains that exhibited glutathione depletion and cascade DOX activation for drug resistance reversal to improve cancer therapy (Xiao et al., 2021).

**FIGURE 1:**
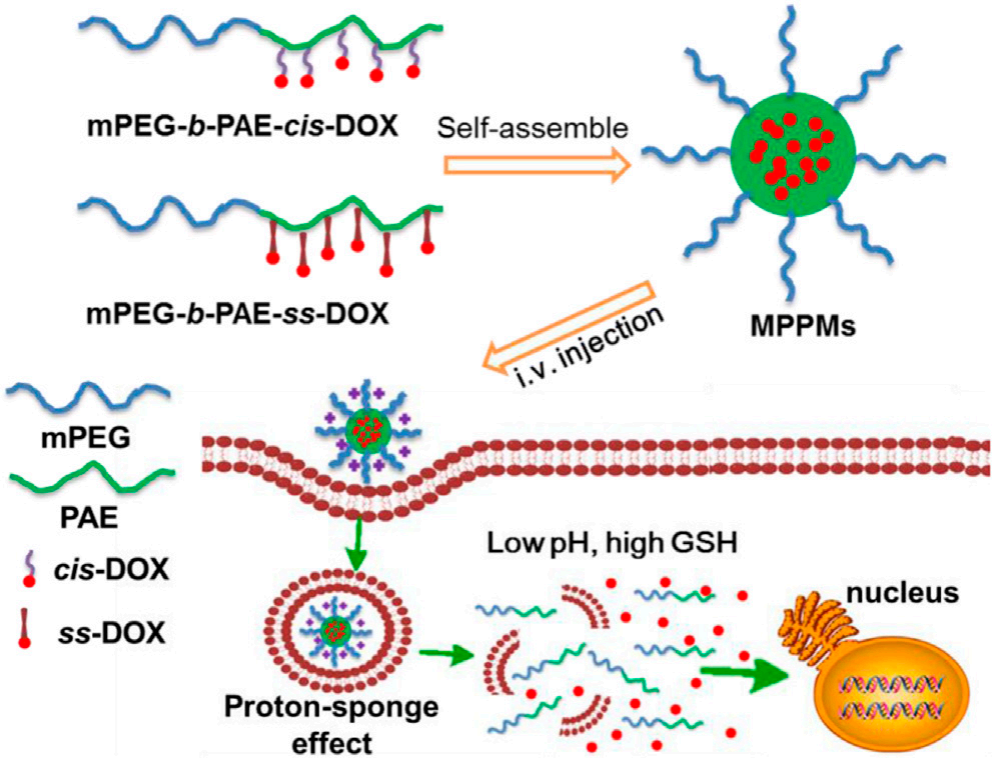
Schematic diagrams of co-micellization of pH/redox dual-responsive polyprodrugs mPEG-*b*-PAE-*cis*-DOX and mPEG-*b*-PAE-*ss*-DOX in an aqueous medium and drug release triggered by acidic pH and high glutathione (GSH) level for cancer chemotherapy.

In this work, inspired by the specific acidic pH and high GSH level in TME, we developed a multifunctional system for drug delivery with pH/redox dual-triggered drug release property based on two polyprodrugs: methyl ether poly(ethylene glycol)-*b*-poly(*β*-amino esters) conjugated with DOX *via* pH-sensitive acid-labile *cis*-aconityl moiety (denoted mPEG-*b*-PAE-*cis*-DOX) and methyl ether poly(ethylene glycol)-*b*-poly(*β*-amino esters) conjugated with DOX *via* GSH-sensitive disulfide bond (denoted mPEG-*b*-PAE-*ss*-DOX). As shown in Figure 1, two polyprodrugs can self-assemble into mixed polyprodrug micelles (MPPMs) structure with high serum stability and prolonged circulation time due to the hydrophilic and nonimmunogenic mPEG shell on the surface (Zhang et al., 2012). Meanwhile, the PAE/DOX core could be protected well during the biological circulation at normal physiological environment. When the MPPMs deposited at the tumor site, PAE segment would be ionized because of low pH (acid), and the solubility would be transferred from hydrophobic to hydrophilic, while the disulfide bonds and *cis*-aconityl moieties between DOX molecules and diblock copolymers would be broken due to high GSH concentration and acidic pH, respectively, resulting in disassembly of micelle and release of cargos, which can induce the apoptosis of tumor cells. The physicochemical properties of MPPMs at different mass ratios, including particle size, zeta-potential, polydispersity index, stability, pH/redox sensitivity, drug release profiles, cytotoxicity *in vivo*, and therapeutic efficacy *in vivo*, would be thoroughly evaluated by a variety of experimental techniques in order to confirm that this formulation could be a promising nanomedicine for cancer chemotherapy.

## Materials and Methods

### Material

Polyprodrugs poly(ethylene glycol) methyl ether-*b*-poly (*β*-amino esters) conjugated with doxorubicin (DOX) *via* redox-sensitive disulfide bonds (mPEG-*b*-PAE-*ss*-DOX, the DOX conjugation efficacy was about 2.2 per polymer molecule, Supplementary Figure S1) and polyprodrug poly(ethylene glycol) methyl ether-*b*-poly (*β*-amino esters) conjugated with DOX *via* pH-sensitive *cis*-aconityl bonds (mPEG-*b*-PAE-*cis*-DOX, the DOX conjugation efficacy was about 2.5 per polymer molecule, Supplementary Figure S1) were synthesized as reported in our previous works (Huang et al., 2018; Zhang et al., 2020). N,N-Dimethylformamide (DMF), dimethyl sulfoxide (DMSO), dichloromethane (DCM), pyrene (99%), and other chemical agents were purchased from Sigma-Aldrich Chemical Co. DL-Dithiothreitol (DTT, 99%), a thiol-based reducing agent, is commonly and widely utilized as a prevailing GSH stimulant in drug release experiment and other *in vitro* experiments (Gao et al., 2019; Zheng et al., 2019; Wei et al., 2020). Phosphate-buffered saline (PBS), fetal bovine serum (FBS), methylthiazoltetrazolium (MTT), Dulbecco’s modified Eagle’s medium (DMEM), and other biological agents were purchased from Invitrogen. The tumor cells MDA-MB-231, A549, and murine fibroblast cell line NIH 3T3 were obtained from the American Type Culture Collection (ATCC). All other biological reagents were used as obtained.

### Preparation of MPPMs

The MPPMs were prepared using the dialysis method (Men et al., 2019). In brief, 30 mg of two polyprodrugs at different mass ratios (mPEG-*b*-PAE-*cis*-DOX/mPEG-*b*-PAE-*ss*-DOX = 2:1, 1:1, or 1:2) were dissolved into 30 ml of DMF with stirring for 30 min at room temperature (RT), followed by transferring into a pre-swollen cellulose membrane bag [molecular weight cutoff (MWCO), 3,500 Da], which was immersed into a beaker (1 L). Then, the dialysis process was performed for at least 48 h against deionized (DI) water at RT. The DI water was replaced every 2 h in the first 24 h and then every 12 h. The solution in the dialysis bag was collected and filtered using a 0.45-μm filter. After lyophilization for another 48 h, the red powder MPPMs were obtained and stored at −20°C for future study.

### Critical Micelle Concentration Measurement

The CMC values of three MPPMs were confirmed by the fluorescence probe technique using pyrene as a fluorescence probe (Luo et al., 2019). When the amphiphilic polymer molecules self-assembled into micelles, pyrene was incorporated into the hydrophobic core instead of the outer shell. Briefly, 1 mg of mixed polyprodrugs was dissolved into acetone (1 ml), followed by addition of 10 ml DI water. Then, the acetone was evaporated by stirring overnight in the dark. After that, a series of concentrations (0.0001–0.1 mg/ml) of polyprodrugs solutions were prepared through dilution. Pyrene was dissolved into acetone, followed by evaporation to form a thin film at the bottom of the vial. Finally, pyrene solution was added at a concentration of 6 × 10^–7^ M into polyprodrugs solution. The resulted mixed solution was equilibrated at RT in the dark overnight before measurement. All the samples were measured by a fluorescence spectrophotometer (F-4500, Hitachi, Japan).

### Characterization

The particle size, polydispersity index (PDI), and zeta-potential (surface charge) were monitored by dynamic light scattering (DLS, Malvern Zeta-sizer Nano S, Malvern, United Kingdom). The samples were re-suspended into a 1.0-ml quartz cuvette, and the measurement was performed using a diode laser of 800 nm at 25°C with 90° of scattering angle. For the zeta-potential measurement, the sample was transferred into the cuvette without bubble, followed by inserting into the sample cell. The zeta-potential was measured at 25°C for three times.

The morphology of the MPPMs was determined by transmission electron microscopy (TEM, Hitachi H-7650, Japan). Briefly, the MPPMs solution was dropped onto the copper grids coated with carbon. After removing the deionized water, the sample was observed by TEM with an acceleration voltage of 80 kV.

To investigate the stability of system, the hydrodynamic diameter and distribution of MPPMs after incubation for different times were measured. In brief, 2 mg of MPPMs was re-suspended in 1 ml of PBS (pH 7.4) with 20% FBS. The resulted solution was incubated for 5 days in the dark at 37°C. The particle size and PDI were recorded using DLS everyday as aforementioned. Moreover, to further confirm the stability of MPPMs, 2 mg of MPPMs was re-suspended in PBS (1 ml, pH 7.4) or 5% glucose solution. Then, the original solution was diluted at 1/1, 1/10, 1/100, and 1/1,000 to prepare the samples for DLS measurement.

### pH/Redox Responsiveness

To evaluate the pH/redox sensitivities, the particle size, zeta-potential, and CMC values of MPPMs at different conditions were studied. For the pH-sensitivity study, the MPPMs (1 mg/ml) were re-suspended in PBS at different pH values (5.0, 5.5, 6.0, 6.5, 7.0, 7.4, and 8.0). The resulted solution was incubated for 24 h at 37°C. Then, the samples were measured using DLS as aforementioned. Furthermore, the CMC values of the system at different pH conditions were measured using the same method as mentioned. For the redox-sensitivity study, the MPPMs were re-suspended in PBS (pH 7.4 with or without 10 mM DTT, pH 6.5 with or without 10 mM DTT), followed by incubation for 24 h at 37°C. Then, the samples were recorded using DLS.

### *In Vitro* Release of DOX From MPPMs

The *in vitro* DOX release profiles from MPPMs at different conditions was investigated by the dialysis method, following previous references with few modifications (Zhang et al., 2016; Men et al., 2019; Jiang et al., 2020). Briefly, 6 mg of MPPMs were dispersed in 6 ml of respective PBS in a dialysis bag (MWCO, 3,500 Da) at a concentration of 1 mg/ml. Then, the bag was immersed into a beaker with 44 ml of PBS (pH 7.4, pH 7.4 with 10 mM DTT, pH 6.5, pH 6.5 with 10 mM DTT), followed by placing in a water bath Dissolution Tester (RCZ-8B, TDTF, China) with stirring (110 rpm) at 37°C. At pre-determined time intervals, 1 ml of solution was taken out for UV-Vis spectrophotometry analysis, and 1 ml of fresh PBS was added. Free DOX formulation was used as control. The cumulative release percent (*E*_r_) was calculated according to the following equation:where *m*_DOX_ was the amount of DOX in MPPMs, *V*_e_ was the volume in dialysis bag (6 ml), *V*_0_ was the whole volume of the release media (50 ml), and *C*_i_ was the concentration of DOX in the *i*th sample. The experiments were carried out in triplicate at each condition.

### Cell Culture

The NIH 3T3, A549, and MDA-MB-231 cell lines were cultured according to the standard protocol from the supplier. Briefly, the cells were cultured in fresh DMEM containing 10% (v/v) FBS, 100 U/ml penicillin, and 100 μg/ml streptomycin in a flask, and incubated at 37°C in an incubator containing 5% of CO_2_.

### Confocal Microscopy Study

The cellular uptake of free DOX and MPPMs in MDA-MB-231 cells were confirmed by confocal laser scanning microscopy (CLSM). In brief, MDA-MB-231 cells were grown on culture dishes (1 × 10^5^ cells/well) in DMEM. After 24 h incubation, the medium was replaced with fresh one. The cells were treated with free DOX or three kinds of MPPMs. After incubation for 4 h, the dishes were washed with PBS three times and fixed with 4% formaldehyde, followed by incubating with 4′,6-diamidino-2-phenylindole (DAPI). After washing three times, the sample was imaged by CLSM (Zeiss, LSM 510, Germany).

### Cytotoxicity

To study the cytotoxicity of free DOX and three MPPMs and Table 2 showed formulations against tumor cells *in vitro*, MTT assay was performed according to the previous reports (Wei et al., 2017; Vater et al., 2020). Briefly, the cells were cultured in DMEM as aforementioned. The cells were first seeded into 96-well plates at a density of 1 × 10^4^ cells/well (200 μl) and cultured overnight. After that, the medium was discarded, and the cells were treated with a series of concentration of free DOX and MPPMs formulations. After incubation for 24 h, MTT solution (5 mg/ml, 20 μl/well) was added, and the plates were shaken for 5 min at 150 rpm. Four hours later, the medium was removed, and DMSO (200 μl) was added. The plates were recorded using a microplate reader (Multiskan Spectrum, Thermo Scientific, Finland) at 490 nm. The cell viability was calculated according to the following equation:where *A*_sample_ and *A*_control_ were the absorbance at 490 nm of cells with or without treatment, respectively. *A*_blank_ was the absorbance at 490 nm of well without cells. The test was performed in replicates of six wells.

### Mice

Adult female BALB/c-nu nude mice (5–6 weeks) were purchased from Beijing Vitalriver Experimental Animal Technology Co. Ltd. The mice were maintained in polyethylene cages with stainless steel lids at room temperature with a 12-h light/dark cycle and covered with a filter cap. Animals were fed with food and water *ad libitum*. All of the animal care and study protocols were approved by the Institutional Animal Care and Use Committee (IACUC) at China Medical University. The mice were narcotized using intraperitoneal (i.p.) administration of the mixture of ketamine (110 mg/kg) and xylazine (5 mg/kg) in saline.

### Therapeutic Efficiency Experiment

To evaluate the therapeutic efficacy of MPPMs *in vivo*, female BALB/c-nu nude mice were used as hosts for tumor xenografts. In brief, MDA-MB-231 cells (1 × 10^6^) were subcutaneously inoculated in the right leg of the mouse. When the tumor volume reached about 100 mm^3^, the mice were randomly divided into three groups (*n* = 10). The mice were treated with three formulations (PBS, 4 mg/kg of free DOX formulation or MPPMs, i.v. administration). The tumor volume, body weight, and survival rate were recorded. The tumor volume was measured by Vernier calipers and defined as (the square of width times length)/2.

### Statistical Analysis

The experimental data were presented with in average values, expressed as the mean ± standard deviation (SD). Statistical analysis was conducted using two-sample Student’s *t*-test of origin 8.5, and considered to be significant when *p* < 0.05.

## Results and Discussion

### Preparation and Characterization of MPPMs

**FIGURE 2:**
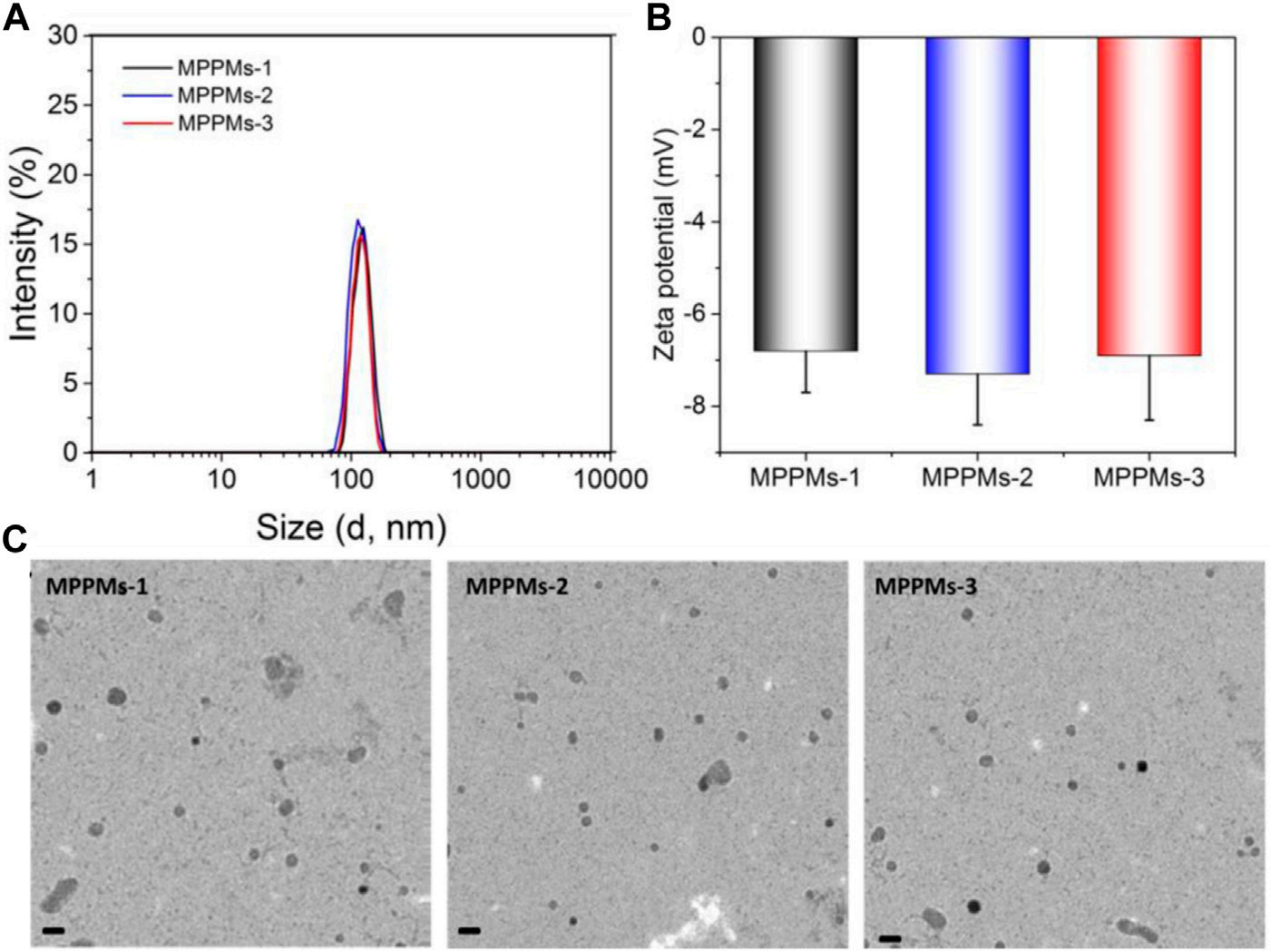
Particle size **(A)**, zeta-potential **(B)**, and TEM images **(C)** of MPPMs self-assembled from two polyprodrugs at different mass ratios. Scale bar: 200 nm *n* = 3, mean ± SD.

The two kinds of polyprodrugs (pH-sensitive mPEG-*b*-PAE-*cis*-DOX and GSH-responsive mPEG-*b*-PAE-*ss*-DOX; chemical structures are shown in Supplementary Figure S1) could self-assemble into MPPMs at different mass ratios (Table 1). The micellar shell was formed by hydrophilic segment mPEG, and the hydrophobic core was constructed with pH-sensitive PAE segment and chemical drug DOX molecules. The particle size, PDI, and zeta-potential of MPPMs were measured by DLS, as shown in Figure 2 and Supplementary Figure S2. The particle size of MPPMs-1 (mPEG-*b*-PAE-*cis*-DOX/mPEG-*b*-PAE-*ss*-DOX = 2:1) was approximately 128 nm. When the mass ratio of mPEG-*b*-PAE-*ss*-DOX was increased, the particle size of MPPMs-2 (mPEG-*b*-PAE-*cis*-DOX/mPEG-*b*-PAE-*ss*-DOX = 1:1) was slightly decreased to about 117 nm. When this mass ratio was 1:2, the particle size of MPPMs-3 was about 125 nm (Figure 2A and Supplementary Figure S2). Furthermore, the morphologies of MPPMs-1, MPPMs-2, and MPPMs-3 were imaged by TEM (Figure 2C), showing that the MPPMs had similar particle size with spherical morphology. The particle size was slightly smaller compared with the result of DLS due to the shrinking of the micelles during dry process for the preparation of sample in TEM analysis. The PDI values of MPPMs-1, MPPMs-2, and MPPMs-3 were, respectively, 0.185, 0.201, and 0.243 (Supplementary Figure S2), indicating that these MPPMs could be uniformly dispersed in water. The zeta-potential of three MPPMs were −6.8, −7.3, and −6.9 mV (Figure 2B), respectively, which were all slightly negative partially due to the PEG shell. PEG shell was widely used to confer a neutral surface charge and stabilize nanoparticles, suggesting that the MPPMs showed high biocompatibility. This core/shell structure with slightly negative charge and PEG shell was able to make MPPMs have high stability and low interaction with serum proteins, and DOX molecules could be protected well during the blood circulation (Feng et al., 2016; Xu et al., 2020). In summary, the MPPMs self-assembled from two kinds of polyprodrugs showed reasonable particle size with spherical morphology, good uniformity, and negative surface charge, which could be a promising drug delivery system for cancer chemotherapy.

**TABLE 1:** The formulation of three MPPMs.

Sample	mPEG-*b*-PAE-*cis*-DOX^a^	mPEG-*b*-PAE-*ss*-DOX^a^
MPPMs-1	2	1
MPPMs-2	1	1
MPPMs-3	1	2

a Mass ratio.

### Self-Assembly and Stability

**FIGURE 3:**
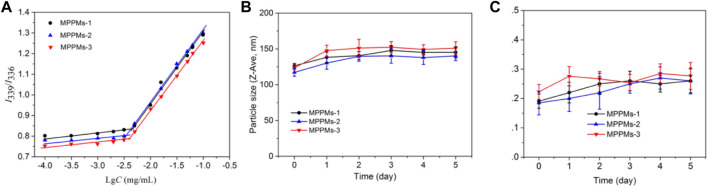
Plot of intensity ratios *I*_339_/*I*_336_ as a function of logarithm of polyprodrugs concentration at pH 7.4 **(A)**. Hydrodynamic diameter **(B)** and polydispersity index (PDI) **(C)** of MPPMs after incubation in PBS at pH 7.4 containing 20% FBS at 37°C for 5 days confirmed by DLS (*n* = 3, mean ± SD).

To investigate the self-assembly of mixed system, the CMC values were measured using fluorescence spectrometry with pyrene as a fluorescence probe. Pyrene was preferentially entrapped into the micellar core during the micellization of system through hydrophobic interaction, whereas it was dispersed into the solution (Basu Ray et al., 2006). Figure 3A represents the change in intensity ratio of *I*_338_/*I*_335_ as function of polyprodrug concentration in aqueous medium at pH 7.4. After analysis of curve fitting, the CMC values of three systems were, respectively, 4.5 μg/ml for MPPMs-1, 3.6 μg/ml for MPPMs-2, and 4.1 μg/ml for MPPMs-3. The low CMCs indicated that the mixed system was able to self-assemble into micelles and exhibited high stability, implying the potential of MPPMs in drug delivery. As reported, the drug delivery system with reasonable size should have high serum stability in order to acquire extended blood circulation time for improving the accumulation at tumor site through the EPR effect (Maeda et al., 2013; Saw et al., 2017). Therefore, the serum stability of mixed systems was further studied. The particle size and PDI of three MPPMs after incubation for different time in PBS (pH 7.4) with 20% FBS at 37°C were monitored by DLS, as shown in Figures 3B,C. The particle size of MPPMs was slightly increased from about 120 nm to approximately 150 nm after incubation for 5 days (Figure 3B), demonstrating the high serum stability of mixed systems. Furthermore, the PDI values of three systems showed negligible changes after incubation for 5 days (Figure 3C), showing the good uniformity and high serum stability of system. In addition, the particle size of three MPPMs in PBS at pH 7.4 or 5% glucose solution showed no significant changes after dilution by 1,000 times (Supplementary Figure S3), further indicating the high stability of three mixed systems. These findings proved that mixed polyprodrugs system were able to self-assemble into micelles (MPPMs) with high serum stability at low concentration, implying that the MPPMs systems could be a promising nanomedicine with prolonged circulation time.

### Dual pH/Redox Responsiveness

**FIGURE 4:**
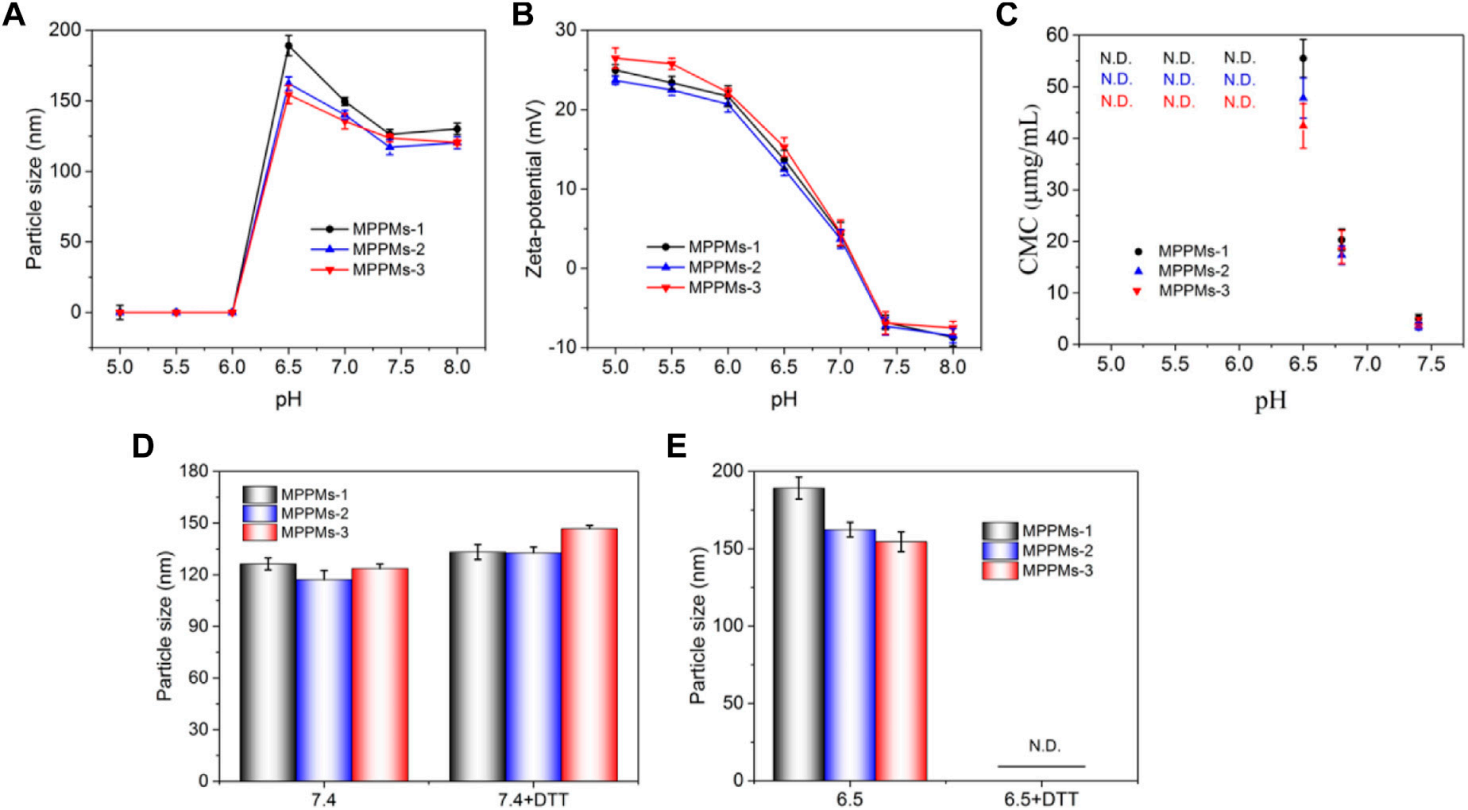
Particle size **(A)** and zeta-potential **(B)** of MPPMs-1, MPPMs-2, and MPPMs-3 in PBS with different pH values after incubation for 4 h. CMC values **(C)** of three MPPMs systems dependent on pH values. Particle size of MPPMs-1, MPPMs-2, and MPPMs-3 in PBS at different conditions: **(D)** pH 7.4 with or without 10 mM DTT and **(E)** pH 6.5 with or without 10 mM DTT) after incubation for 4 h (*n* = 3, mean ± SD).

Next, the pH/redox dual-responsiveness of MPPMs was investigated, as shown in Figure 4. After incubation in PBS at different pH values, the particle size and zeta-potential of MPPMs are shown in Figures 4A,B, respectively. When the pH decreased from 8.0 to 6.5, the particle size increased from about 125 to 180 nm. The reason could be that the solubility transformation of PAE from hydrophobicity to hydrophilicity caused by the protonation of tertiary amine residues in PAE segments in acidic environment, leading to the swelling of MPPMs. When the pH was lower than 6.5, all tertiary amine residues in PAE segments were protonated, and the pH-sensitive *cis*-aconityl linkers were simultaneously broken, resulting in disassembly of MPPMs systems. Therefore, no nanoparticle was recorded by DLS (Figure 4A). In addition, compared with MPPMs-2 and MPPMs-3, MPPMs-1 showed greater rangeability (from 126.3 to 189.2 nm) because of the highest mass ratio of mPEG-*b*-PAE-*cis*-DOX in the mixed system. When the pH decreased from 8.0 to 5.0, the zeta-potential of MPPMs obviously increased (ca*.* −7 mV at pH 8.0 to + 25 mV at pH 5.0, Figure 4B), resulting from the ionization of tertiary amine residues in PAE segment and the acidification of separate DOX molecules in the solution. These results proved the pH-sensitivity of MPPMs. Additionally, the charge reversal of MPPMs from negative to positive charge can enhance the cellular uptake. Moreover, the CMC values of MPPMs systems at different pH conditions were measured, as shown in Figure 4C. For MPPMs-1, the CMC value was increased from 4.5 to 55.3 μg/ml when the pH decreased from 7.4 to 6.5, resulting from the solubility transformation of PAE segment caused by protonation of tertiary amine residues. Because the molecular polarity was steadily enhanced, it required a stronger driving force to counteract the increased electrostatic repulsive force for micellization of system, when the PAE segments in the system were transferred from hydrophobic to hydrophilic. Additionally, the increase in surface charge would further enhance the repulsive force, which required greater driving force to offset that. When the pH was lower than 6.5, the CMC was not detectable, attributing to complete protonation of tertiary amine residues and cleavage of *cis*-aconityl bonds. Similar change trends were found for MPPMs-2 and MPPMs-3 (Figure 4C). Particularly, with the increase in mass ratio of polyprodrug mPEG-*b*-PAE-*ss*-DOX, the CMC of mixed system was decreased. All the findings demonstrated the pH sensitivity of three MPPMs systems. To investigate the redox responsibility of mixed system, the particle size of MPPMs after incubation in PBS at pH 7.4 or 6.5 with or without DTT (10 mM) was recorded by DLS, as shown in Figures 4D,E. The particle sizes of three MPPMs slightly increased after incubation in PBS at pH 7.4 with 10 mM DTT compared with those at pH 7.4 without DTT (Figure 4D), while no particle was observed after incubation in PBS at pH 6.5 with 10 mM DTT (Figure 4E). At pH 7.4 with DTT (10 mM), the disulfide bonds conjugated DOX molecules with PAE segments in polyprodrug mPEG-*b*-PAE-*ss*-DOX were broken, thereby leading to the slight swelling of micelles. By the way, with the increase in mass ratio of mPEG-*b*-PAE-*ss*-DOX in the mixed system, the MPPMs-3 showed the biggest difference in size compared with MPPMs-1 and MPPMs-2. At pH 6.5 without DTT, the particle sizes of three MPPMs increased obviously in comparison to those at pH 7.4 due to swelling of polyprodrug micelles induced by protonation of tertiary amine groups and the cleavage of *cis*-aconityl bonds. By contrast, at pH 6.5 with DTT, the protonation of tertiary amine residues in PAE segment and cleavage of chemical bonds (disulfide bonds in mPEG-*b*-PAE-*ss*-DOX and *cis*-aconityl bonds in mPEG-*b*-PAE-*cis*-DOX) caused the disassembly of mixed system, resulting in non-detection of particles (Figure 4E). In summary, the prepared MPPMs exhibited pH sensitivity and could respond to the high reducing agent concentration that could be used for anticancer controlled release.

### pH/Redox-Triggered Drug Release Profile

**FIGURE 5:**
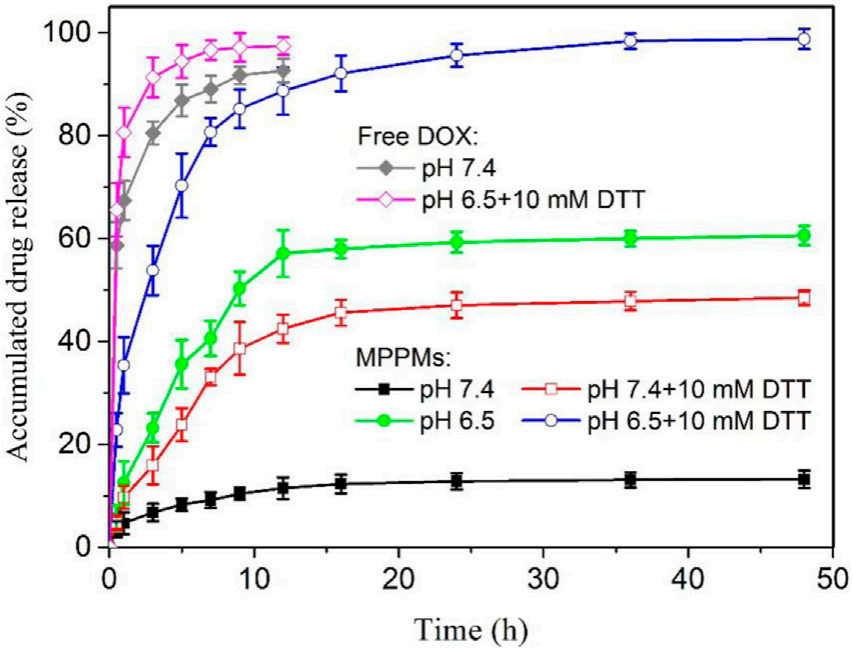
The *in vitro* DOX release profiles of MPPMs-1 in PBS at different conditions (pH 7.4, pH 6.5, pH 7.4 with 10 mM DTT and pH 6.5 with 10 mM DTT). The release profiles of free DOX at pH 7.4 and 6.5 with DTT (10 mM) were used as control (*n* = 3, mean ± SD).

Since the pH/redox dual responsibility of MPPMs has been evaluated, the *in vitro* DOX release profiles from MPPMs at different conditions were next investigated using the dialysis method. Different conditions were selected to simulate the normal physiological condition (PBS, pH 7.4) and tumor microenvironment (PBS, pH 6.5 with 10 mM DTT). Free DOX formulation was used as control. As shown in Figure 5, the drug release rate of free DOX was rapid. The cumulative release amount of free DOX was higher than 90% for 12 h in PBS at pH 7.4 or pH 6.5 with 10 mM of DTT. By contrast, at pH 7.4, the DOX release rate of MPPMs-1 was much lower, and the cumulative release amount of DOX was <15% for 48 h, suggesting that the cargos were protected well in the micellar core. When the pH decreased to acidic condition (pH 6.5), the drug release rate was obviously accelerated, and the accumulated drug release was higher than 50% for 12 h and approximately 60% for 48 h, respectively. The reason could be that the pH-sensitive *cis*-aconityl bonds between DOX molecules and PAE segments in polyprodrug mPEG-*b*-PAE-*cis*-DOX were broken, and the DOX molecules were released from the micelles. In the presence of DTT (10 mM) at pH 7.4, the DOX release rate was much higher compared with that at pH 7.4 without DTT. Additionally, the cumulative drug release was about 40% for 12 h and 48% for 46 h, respectively, resulting from the cleavage of redox-responsive disulfide bonds in polyprodrug mPEG-*b*-PAE-*ss*-DOX. At pH 6.5 with 10 mM DTT, the DOX release rate was dramatically accelerated in comparison to that at pH 6.5 without DTT, and the accumulated DOX release was higher than 85% for 12 h and approximately 100% for 48 h, resulting from the cleavage of *cis*-aconityl and disulfide bonds in the mixed system. Moreover, the release rate and cumulative drug release of MPPMs at pH 6.5 were slightly higher in comparison to those at pH 7.4 with DTT. The reason could be that the mass ratio of mPEG-*b*-PAE-*cis*-DOX and mPEG-*b*-PAE-*ss*-DOX was 2:1 in MPPMs-1 system, displaying that more DOX molecules were released due to the cleavage of pH-sensitive *cis*-aconityl bonds. As expected, when the mass ratio of polyprodrug mPEG-*b*-PAE-*ss*-DOX increased to 1:1 (MPPMs-2) and 1:2 (MPPMs-3), the accumulated DOX release for 48 h at pH 7.4 with DTT was enhanced (50% for MPPMs-2 and 70% for MPPMs-3), as shown in Supplementary Figure S4. It’s interesting to find that the drug release profiles could be regulated by changing the mass ratios of two polyprodrugs. Collectively, the release rate and accumulated release amount of drug from MPPMs were significantly influenced by the pH value and DTT concentration, suggesting the cargos could be controlled release from the MPPMs “on-demand.”

### Cytotoxicity Assay

**FIGURE 6:**
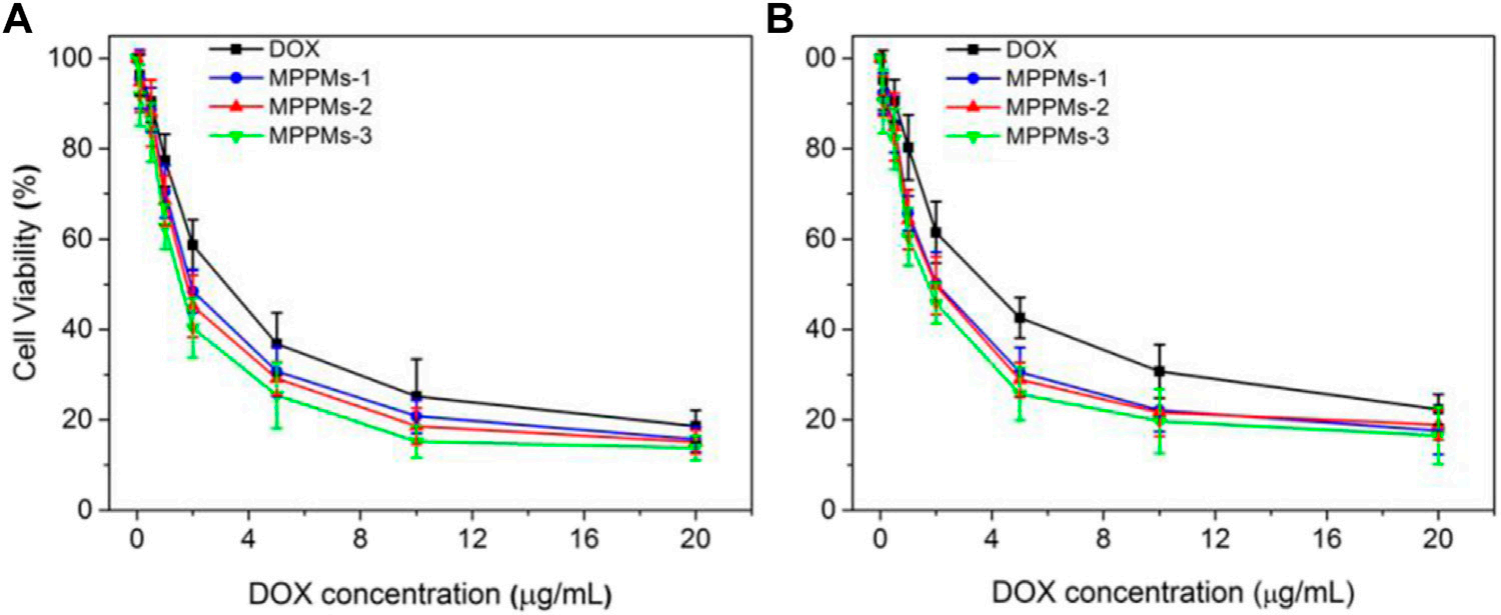
The cytotoxicity for A549 **(A)** and MDA-MB-231 **(B)** cells treated with free DOX, MPPMs-1, MPPMs-2, and MPPMs-3 formulation for 24 h in concentration specified (*n* = 6, mean ± SD).

Since the mixed polyprodrug micelles have been successfully prepared and the pH/redox-triggered drug release performance has been confirmed, the cytotoxicity of three MPPMs against tumor cells (A549 and MDA-MB-231) was next evaluated using MTT. The cytotoxicity of blank diblock copolymer mPEG-*b*-PAE against NIH 3T3 cells was first studied, as shown in Supplementary Figure S5. With increase in copolymer concentration, the toxic effect was slightly increased. However, even at the highest concentration of 400 μg/ml, the cell viability of NIH 3T3 was still higher than 85%, indicating the negligible cytotoxicity and high biocompatibility of diblock copolymer mPEG-*b*-PAE, which was used as carrier in this work. Figure 6 and Table 2 showed the cytotoxicity of free DOX and three MPPMs against tumor cells A549 and MDA-MB-231 for 24 h in DOX concentration gradients. As expected, with increase in DOX concentration, the cell viability of A549 and MDA-MB-231 was sharply decreased. Compared with free DOX, three MPPMs systems showed higher toxic effect against A549 and MDA-MB-231 cells. For A549 cell (Figure 6A), the IC50 values were, respectively, 2.44 μg/ml for free DOX, 1.98 μg/ml for MPPMs-1, 1.95 μg/ml for MPPMs-2, and 1.81 μg/ml for MPPMs-3. For MDA-MB-231 cell (Figure 6B), the IC50 values were 3.95 μg/ml for free DOX, 2.06 μg/ml for MPPMs-1, 1.93 μg/ml for MPPMs-2, and 1.90 μg/ml for MPPMs-3, respectively. In summary, the diblock copolymer showed negligible toxic effect, whereas the three MPPMs were able to efficiently kill the tumor cells compared with free DOX, indicating that the MPPMs could deliver DOX molecules into tumor cells.

**TABLE 2:** The IC50 values of three MPPMs against A549 and MDA-MB-231 cells.

Cell	Free DOX	MPPMs-1	MPPMs-2	MPPMs-3
A549	2.44 μg/ml	1.98 μg/ml	1.95 μg/ml	1.81 μg/ml
MDA-MB-231	3.95 μg/ml	2.06 μg/ml	1.93 μg/ml	1.90 μg/ml

### Cellular Uptake

**FIGURE 7:**
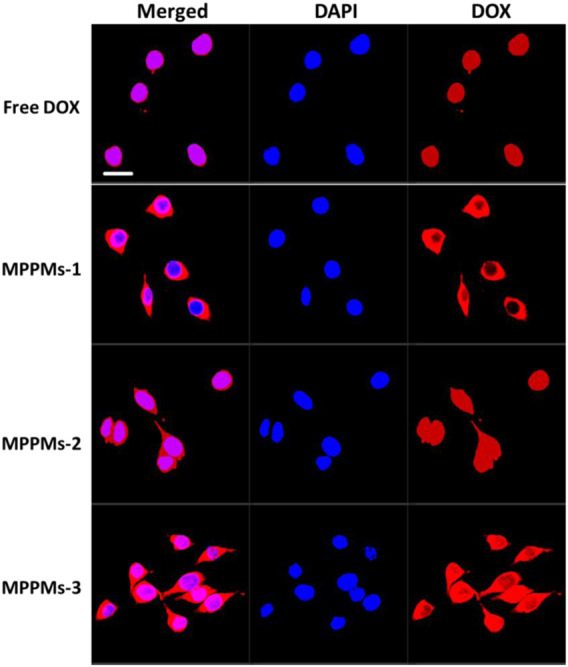
CLSM images of MDA-MB-231 cells incubated with free DOX for 4 h. Merged images were overlap of DAPI (blue) and DOX (red). Scale bar: 20 μm.

Next, the cellular uptake and distribution of free DOX and three MPPMs after incubation with MDA-MB-231 cells for 4 h was studied by confocal laser scanning microscopy (CLSM), as shown in Figure 7. For free DOX formulation, almost all the DOX molecules can quickly enter the tumor cell and co-locate with cell nucleus region at 4 h, due to small molecule property of free DOX. For the MPPMs-1, although the DOX can enter the tumor cell quickly, most of DOX molecules distributed in the cytoplasm. The MPPMs-2 and MPPMs-3 can effectively deliver the DOX molecules to the cell nucleus compared with MPPMs-1. The free DOX formulation can deliver drug molecules to the cell nucleus better than MPPMs, resulting from the small chemical drug molecules entering the tumor cell and depositing into the cell nucleus quickly. For the MPPMs, the nanoparticles should bind on the surface and enter the tumor cell through endocytosis and other ways, followed by responding to the acidic pH and high GSH level cues to release the cargos (Figure 5 and Supplementary Figure S6). In summary, the three MPPMs can deliver the DOX molecules to the tumor cells efficiently and the free DOX formulation *in vitro*, especially MPPMs-2 and MPPMs-3, showing the similar antitumor activity of MPPMs compared with free DOX. In the future, MPPMs-2 would be selected for the animal experiment to evaluate the therapeutic efficacy and side effect.

### Therapeutic Efficacy and Biosafety *In Vivo*

**FIGURE 8:**
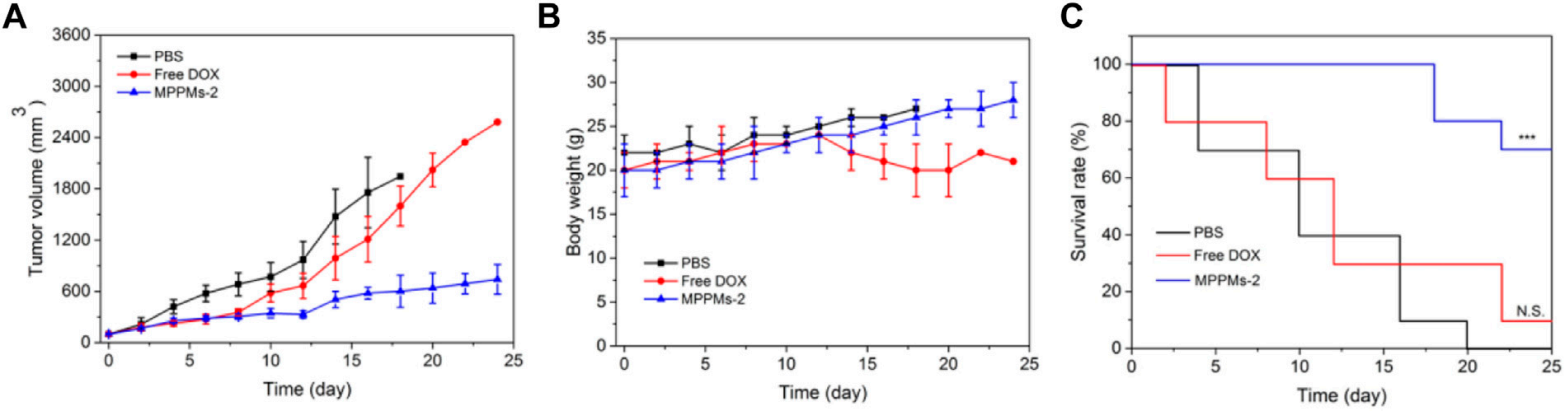
**(A)** Tumor volume of tumor-bearing mice after different PBS, free DOX, and MPPMs-2 treatments. **(B)** The body weight of tumor-bearing mice after different PBS, free DOX, and MPPMs-2 treatments (*n* = 10, mean ± SD). **(C)** Survival rates of tumor-bearing mice after different PBS, free DOX, and MPPMs-2 treatments. Statistical analysis was done using Kaplan–Meier method (*n* = 10). *p* values: *p < 0.05, **p < 0.01, ***p < 0.001; N.S., no significance.

Since the pH/redox dual responsibility, drug release profile, cytotoxicity, and intracellular uptake of MPPMs have been investigated, we next evaluated the tumor therapy efficacy *in vivo*. The MDA-MB-231 cells were inoculated on the right leg of mice. When the tumor size was approximately 100 mm^3^, the tumor-bearing mice were randomly divided into three groups: PBS, free DOX (4 mg/kg), and MPPMs-2 (equal to 4 mg/kg of DOX). Different drug formulation was intravenously (i.v.) administrated through the tail vein. The tumor volume, tumor inhibitory rate, body weight, and survival rate of tumor-bearing mice with different treatments were recorded, as shown in Figure 8 and Supplementary Figure S7. For the free DOX treatment, the tumor was inhibited first, and the tumor volume grew slowly. However, the tumor grew quickly after the 10th day similar to the PBS treatment group, possibly resulting from the drug resistance and side effect. By contrast, the tumor volume of tumor-bearing mice treated with MPPMs-2 was effectively inhibited compared with PBS and free DOX treatments (Figure 8A; Supplementary Figure S7). The body weight of mice treated with PBS, free DOX, and MPPMs-2 was recorded, as shown in Figure 8B. The mice treated with PBS as a control showed negligible changes, while the free DOX treatment group showed obvious body weight loss because of side effect. For the MPPMs, the body weight increased slowly with the treatment time. These results not only demonstrated that the MPPMs can efficiently inhibit the growth of tumor but also can reduce the side effect compared with PBS control and free DOX formulation. Next, the survival rate of mice with different treatments was recorded, as shown in Figure 8C. For the PBS treatment, the survival rate was decreased quickly, and all of the mice were dead at 20th day. For the free DOX treatment, at 24th day, only 10% of tumor-bearing mice was alive. By contrast, the survival rate of MPPMs-2-treated mice group was still higher than 70% even at the 24th day, suggesting the much higher antitumor activity compared with other controls. In addition, the biosafety of MPPMs system was further evaluated. Supplementary Figure S8 shows the weight of major organs (heart, liver, spleen, lung, and kidney) of mice treated with different formulations. We could find that MPPMs-2 showed reduced cardiotoxicity compared with free DOX formulation, which was proved by the weight change of the heart. Moreover, the blood biochemistry analysis was proceeded to study the cardiotoxicity of different formulations, as shown in Supplementary Figure S9. The results showed that the heart function marker (CK) of free DOX-treated group was much higher than that of the normal group, while the CK value of MPPMs-2-treated group was similar to that of the normal group. These results indicated that the cardiotoxicity of DOX molecules was obviously reduced by formulating in MPPMs-2. Other factors such as hepatic function markers (ALT, AST), uric acid (UA), and renal function markers (CREA, BUN) of free DOX-treated group were also increased compared with those of normal and MPPMs-2-treated groups. In summary, the MPPMs-2 showed much lower cardiotoxicity and side effect compared with free DOX formulation, suggesting the high biosafety of MPPMs system. Taken together, the MPPMs-2 can efficiently and effectively inhibit the growth of tumor with high therapeutic efficacy and reduced side effect.

## Conclusion

In the present study, a pH/redox dual-responsive MPPMs system self-assembled from pH-sensitive polyprodrug mPEG-*b*-PAE-*cis*-DOX and redox-responsive polyprodrug mPEG-*b*-PAE-*ss*-DOX at different mass ratios were prepared and used for drug delivery with improved therapeutic efficacy and reduced side effect. These MPPMs exhibited reasonable size and negatively charged surface for drug delivery with PEG shell and PAE/DOX core. The systems also showed quite low CMC values and high serum stability, facilitating the high accumulation of MPPMs at tumor site *via* the EPR effect due to the prolonged circulation time. The particle size and CMC value of MPPMs increased with the decrease in pH. Additionally, the surface charge of MPPMs sharply increased with the decrease in pH. These results proved the pH sensitivity of MPPMs. Furthermore, the particle size of MPPMs was slightly increased at pH 7.4 with DTT compared with that at pH 7.4 without DTT. By contrast, no particle size was detected at pH 6.5 with DTT. These findings demonstrated the redox responsibilities of MPPMs. The *in vitro* drug release experiments showed that the DOX release profile from MPPMs was pH/redox triggered. The MPPMs can efficiently deliver the DOX molecules to the tumor cell nucleus compared with free DOX. The *in vivo* experiment showed that the MPPMs can effectively inhibit the tumor growth with improved therapeutic efficacy and reduced side effect. In summary, the prepared MPPMs could be a potential nanomedicine for cancer chemotherapy. By the way, this MPPMs might be a stimuli-responsive noncarrier for drug delivery. The drug molecules could be physically loaded into the MPPMs for drug delivery and controlled release. In addition, it is inspirational to develop multifunctional systems for extra- and intracellular drug delivery with multi-staged drug release profiles based on different carriers for cancer therapy. However, more efforts have to be made in safety evaluation before it could be widely use in clinics.

## Data Availability

The original contributions presented in the study are included in the article/Supplementary Material. Further inquiries can be directed to the corresponding author.
