# Supplementary material for: Fabrication of pH/Redox Dual-Responsive Mixed Polyprodrug Micelles for Improving Cancer Chemotherapy
Source: Front Pharmacol. 2022 Feb 3;12:802785. doi: 10.3389/fphar.2021.802785 (PMC8850636; doi:10.3389/fphar.2021.802785)
Supplement: Supplementary file 1 [file DataSheet1.doc]

**Fabrication of pH/redox dual-responsive mixed polyprodrug micelles for improving cancer chemotherapy**

Ji Luo^1^, Shuguang Zhang^1^, Peiyao Zhu, Wenke Liu, Jiang Du^*^

Department of Thoracic Surgery, The First Affiliated Hospital of China Medical University, Shenyang 110001, China

^1^J.L. and S.Z. contribute equally to this work.

*Corresponding author:

Jiang Du: E-mail: [jiangdu@cmu.edu.cn](mailto:jiangdu@cmu.edu.cn)

**Figure S1** Chemical structures of polyprodrugs mPEG-*b*-PAE-*cis*-DOX (upper) and mPEG-*b*-PAE-*ss*-DOX (below).


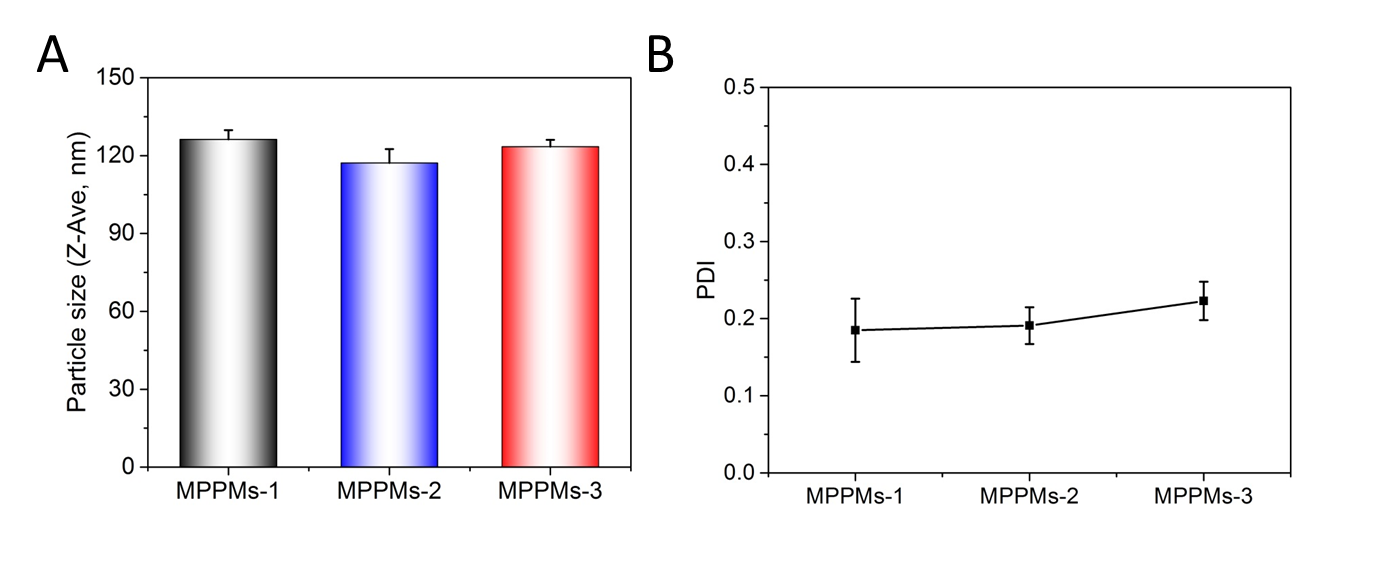


**Figure S2** Particle size (A) and PDI (B) of the MPPMs-1, MPPMs-2 and MPPMs-3 measured by DLS (n= 3, mean ± S.D.).


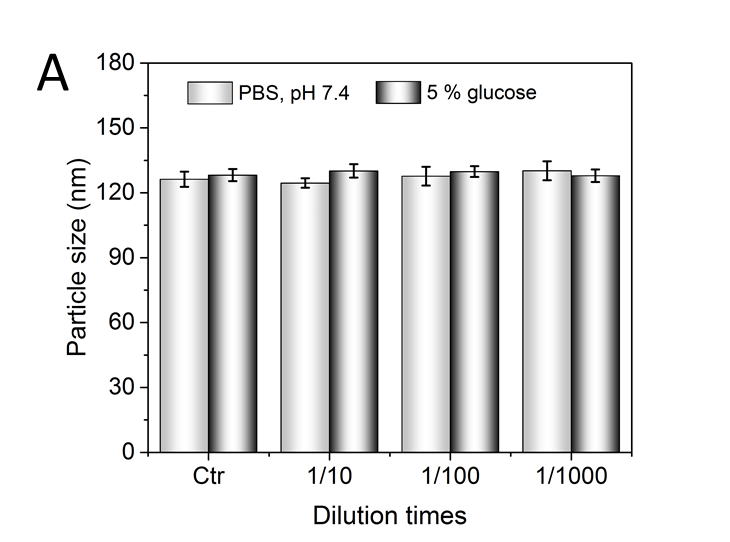


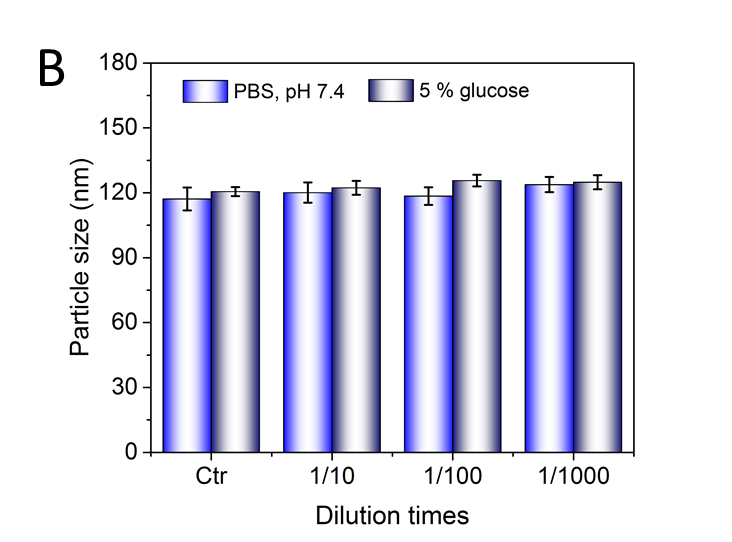


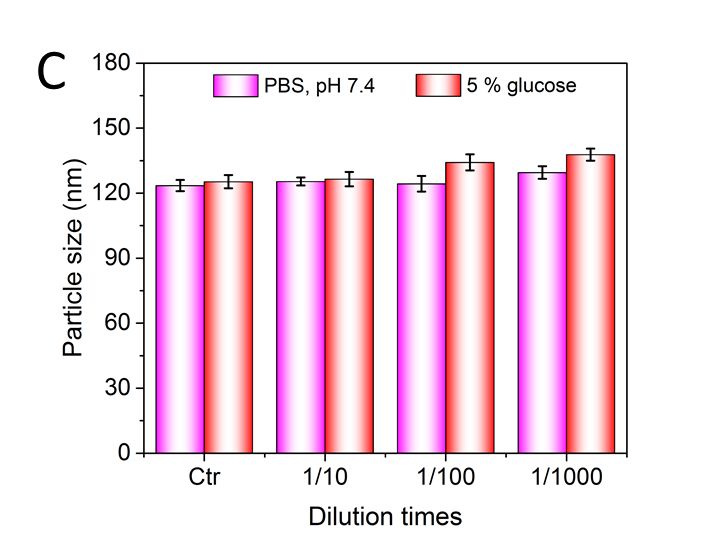


**Figure S3** Particle sizes of the MPPMs-1 (**A**), MPPMs-2 (**B**) and MPPMs-3 (**C**) in PBS at pH 7.4 and 5 % glucose upon dilution at 1/10, 1/100 and 1/1000 of the original concentration of nanoparticles, which was 2 mg/mL (n = 3, mean ± S.D.).


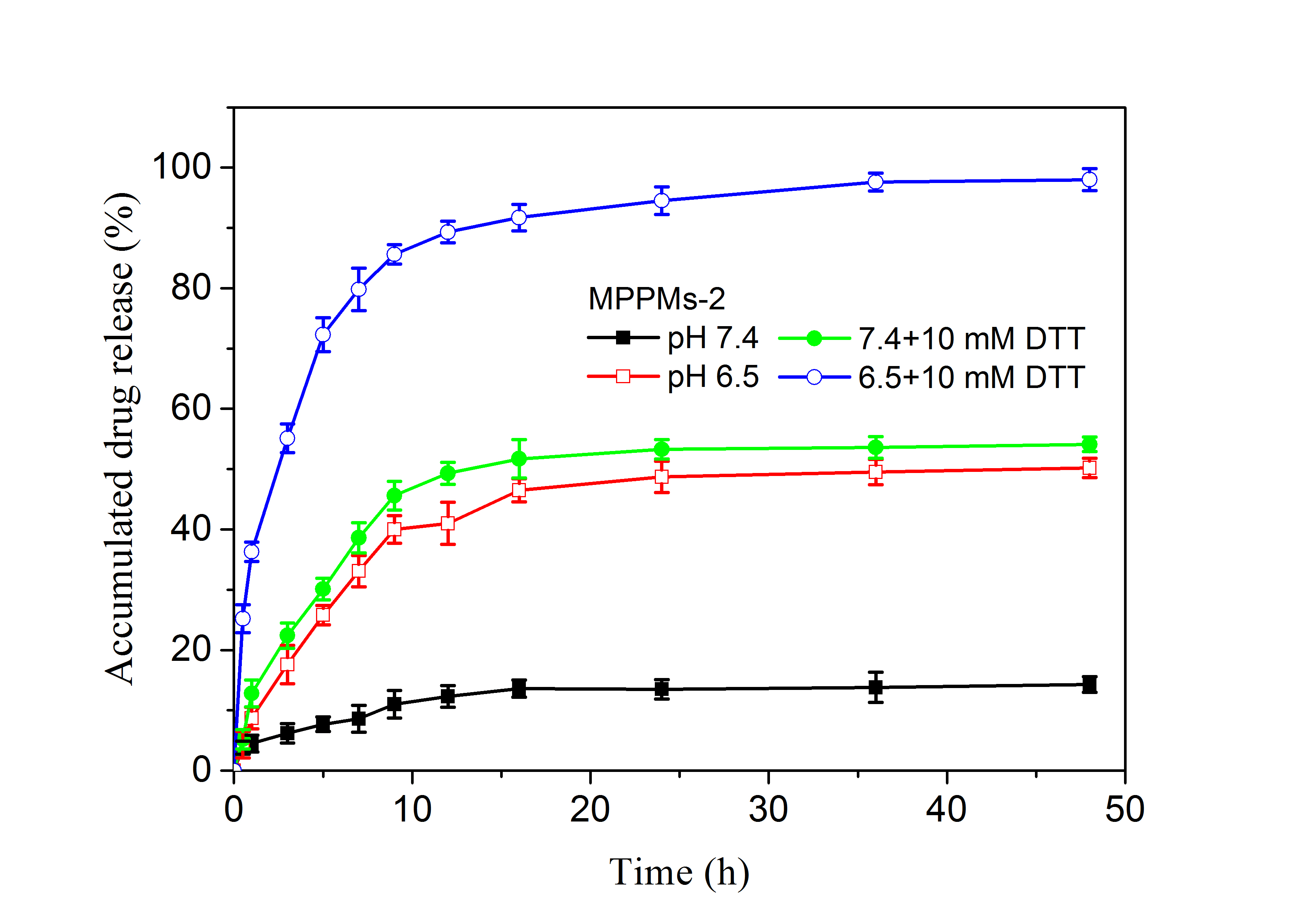

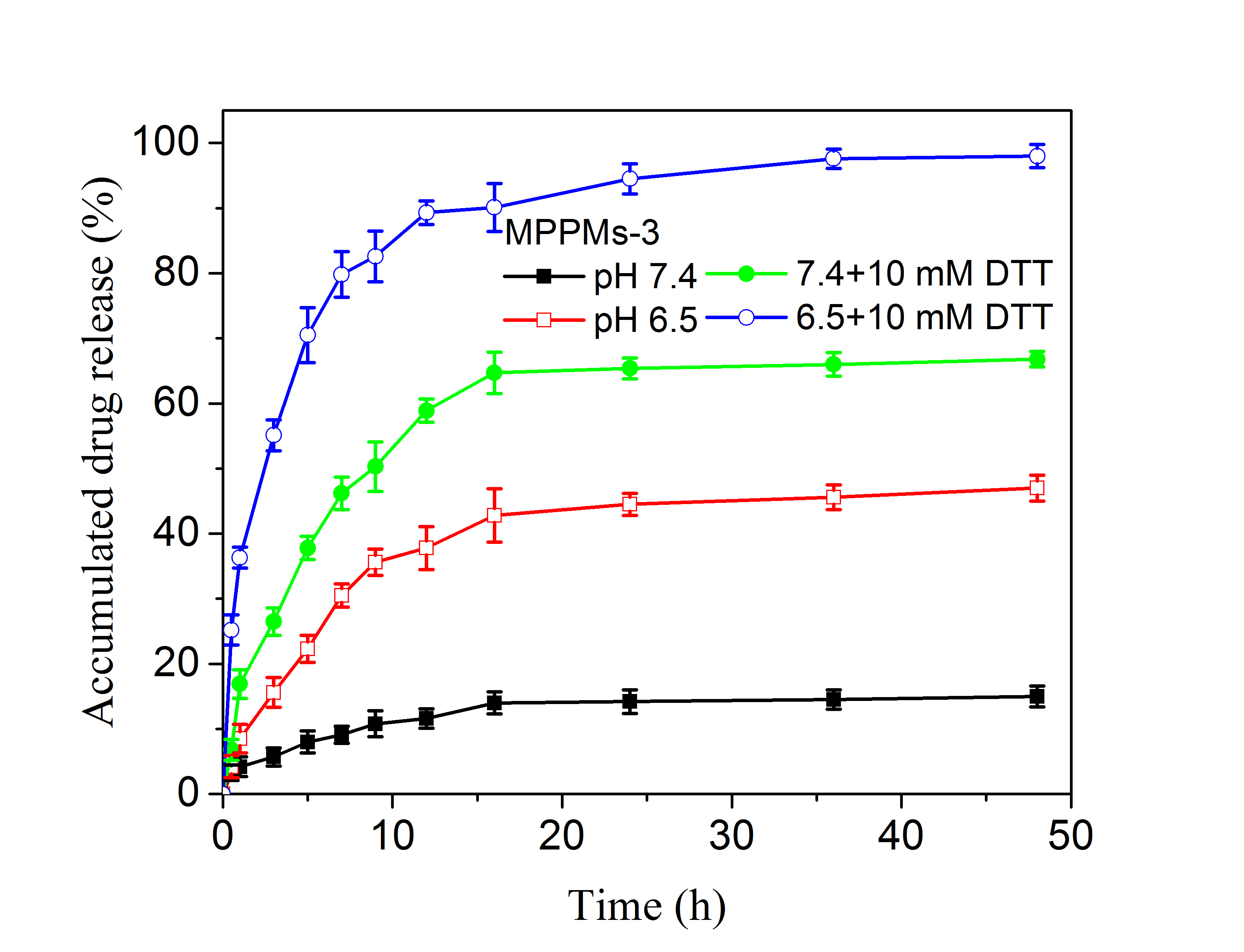


**Figure S4** The *in vitro* DOX release profiles of MPPMs-2 (left) and MPPMs-3 (right) in PBS at different conditions (pH 7.4, pH 6.5, pH 7.4 with 10 mM DTT and pH 6.5 with 10 mM DTT) (n = 3, mean ± S.D.).

**
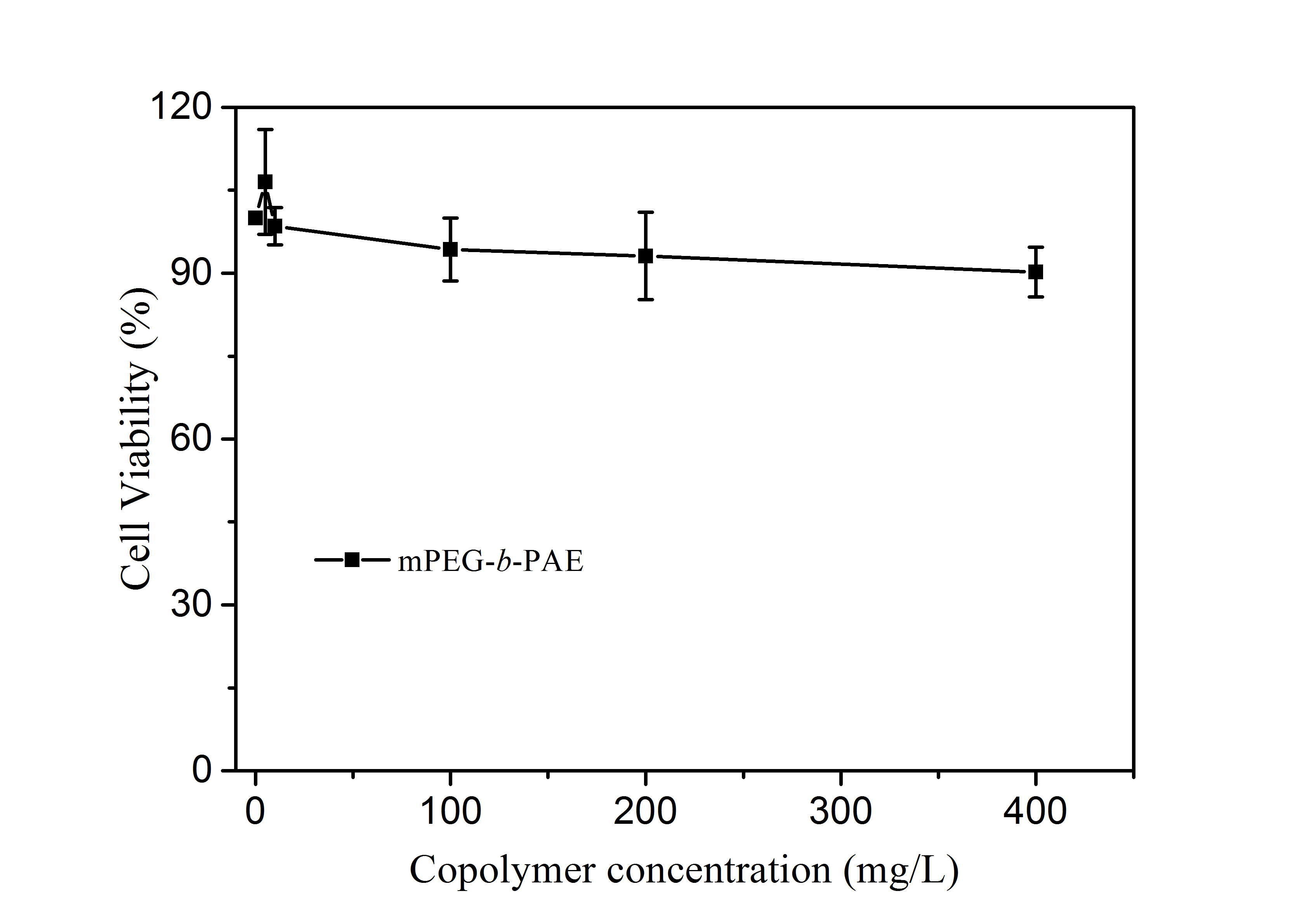
**

**Figure S5** The cytotoxicity of diblock copolymer mPEG-*b*-PAE against NIH 3T3 cells for 24 h in concentration specified.


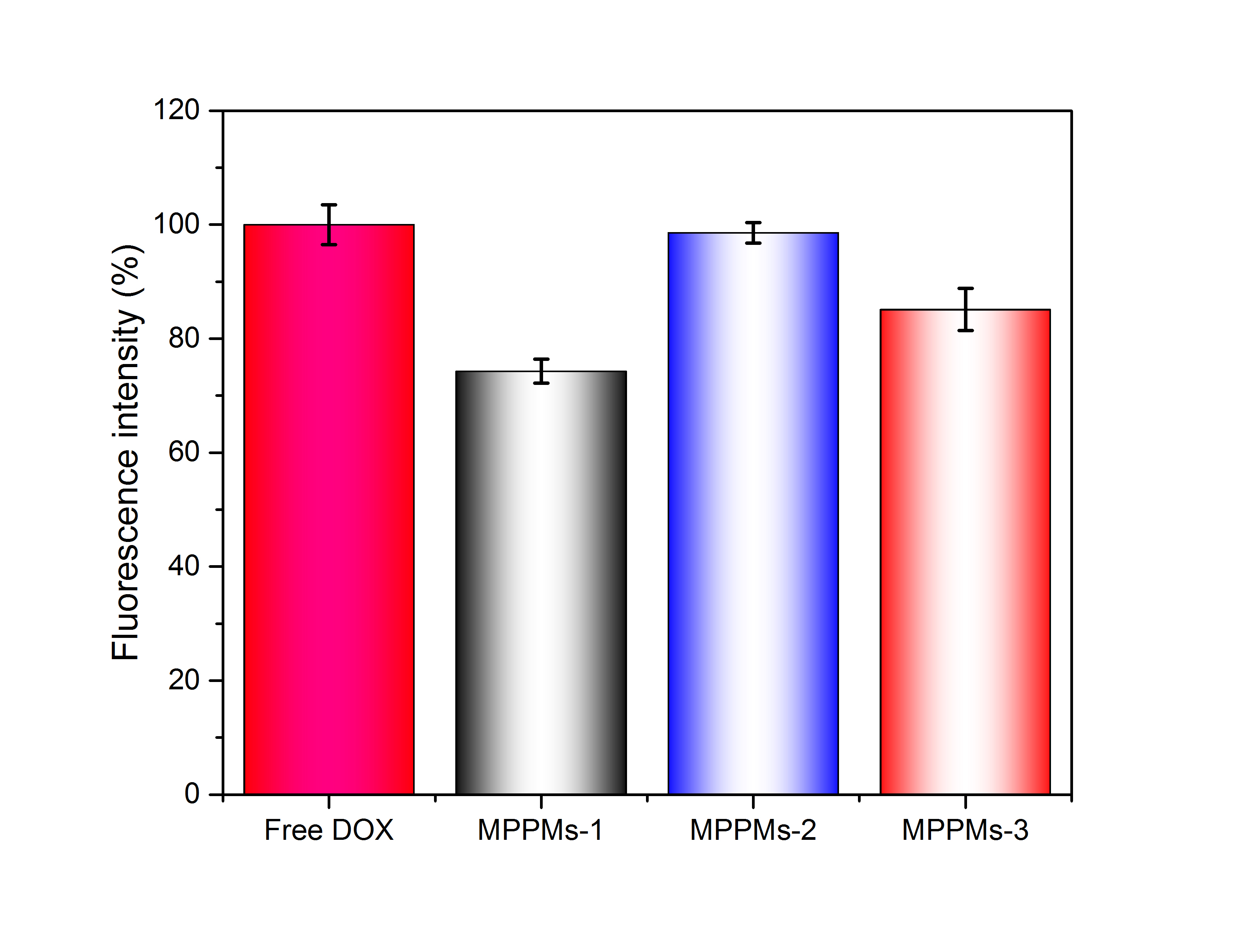


**Figure S6** The fluorescence intensity of free DOX and three MPPMs treated cells confirmed by ImgeJ software based on Figure 7.


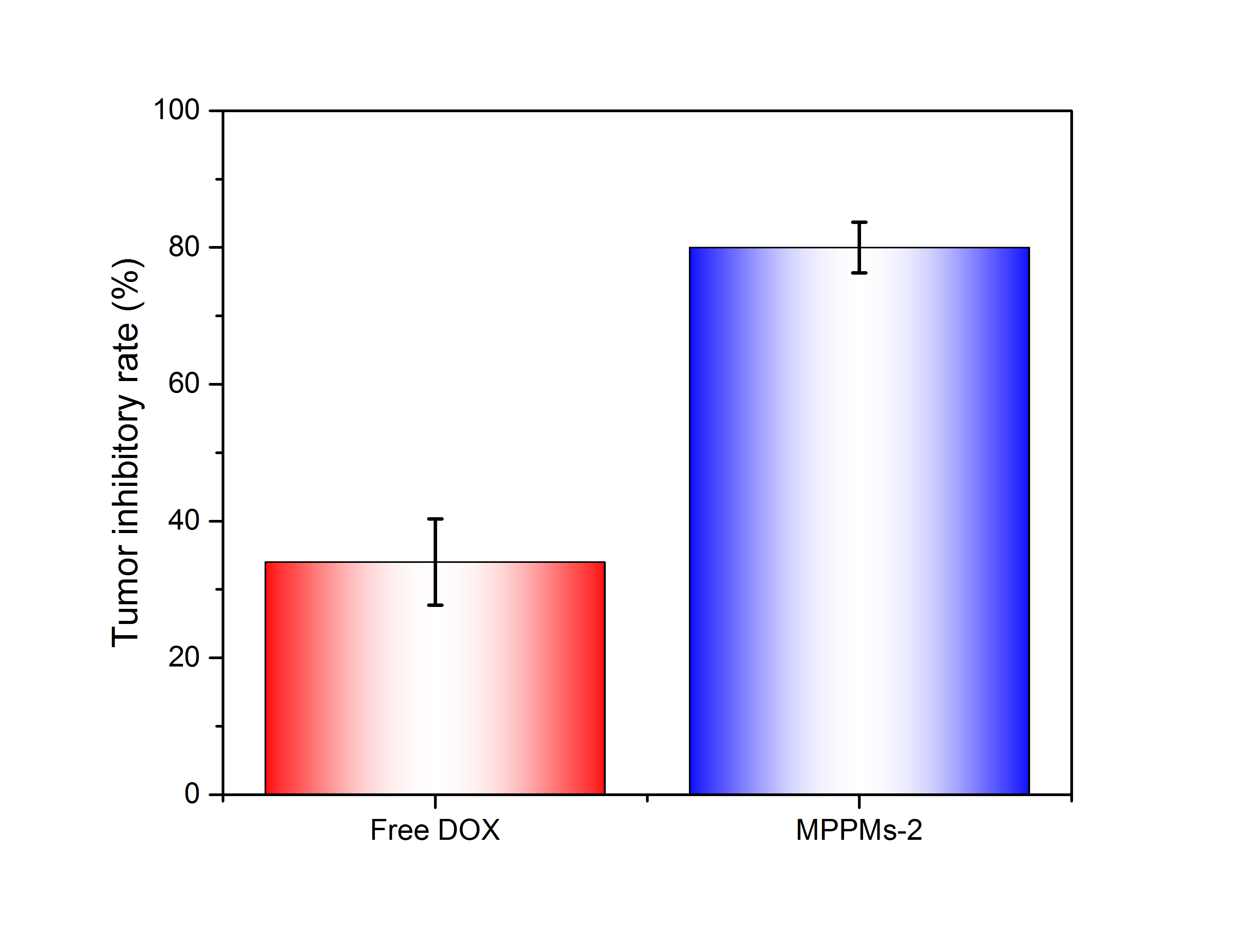


**Figure S7** The tumor inhibitory rate of free DOX and MPPMs-2 at 20-day.


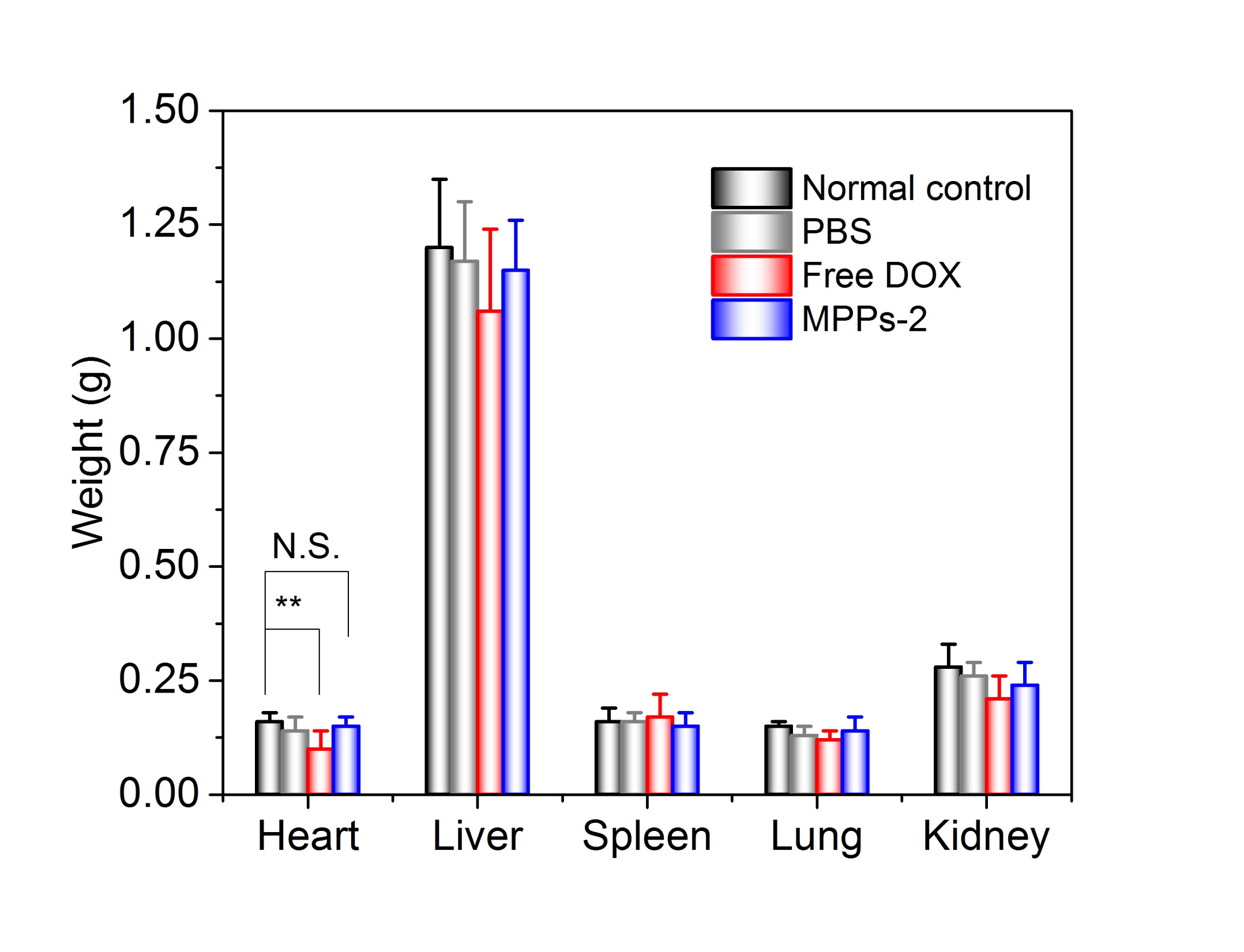


**Figure. S8** The weight of major organs of mice treated with PBS, free DOX, and MPPMs-2, respectively. P values: ***p*<0.01, N.S., no significant difference.


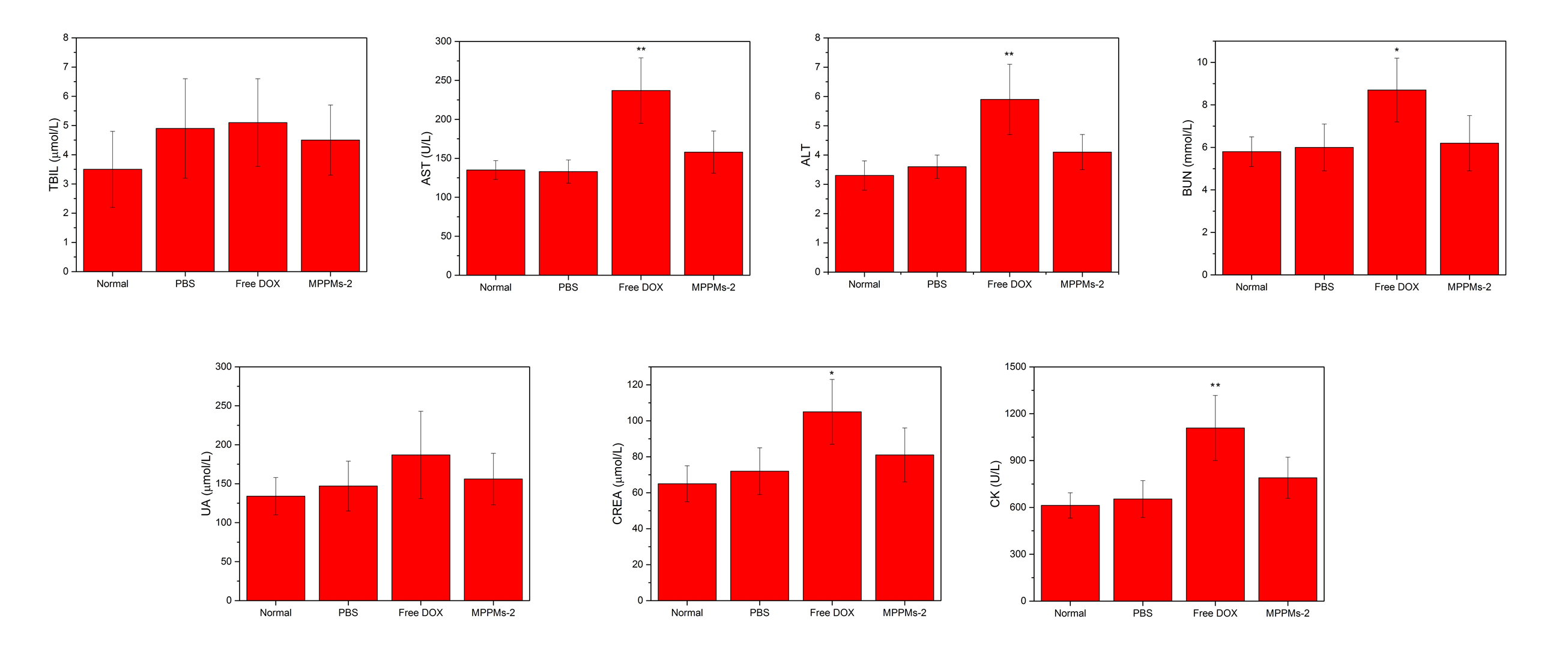


**Figure. S9** Blood biochemistry analysis of the mice treated with PBS, free DOX, and MPPMs-2, respectively. The results show mean and standard deviation of total bilirubin (TBIL), aminotransferase (ALT), aminotransferase (AST), blood urea nitrogen (BUN), uric acid (UA), creatinine (CREA), creatine kinase (CK). P values: **p*<0.05, ***p*<0.01.
